# Bridging the difficult talk: dentists’ perception of a patient-centred communication aid to improve oral cancer early detection

**DOI:** 10.1186/s12903-026-08911-5

**Published:** 2026-06-29

**Authors:** Xuanyun Lu, Gerald Humphris, Jonathon Timothy Newton, Siyang Yuan

**Affiliations:** 1 School of Dentistry, University of Dundee, Dundee, UK; 2 School of Medicine, University of St Andrews, St Andrews, UK; 3 Faculty of Dentistry, Oral & Craniofacial Sciences, King’s College London, London, UK

**Keywords:** Question Prompt List, Communication, Oral Cancer, Dental Care, Consultation, Early Detection, Patient Engagement

## Abstract

**Background:**

Oral cancer survival remains poor in Scotland, which is partly due to the delay of early detection. Patients’ poor awareness contributes to such delays. Dentists often avoid raising the topic of oral cancer during routine check-ups, fearing patient anxiety. Question Prompt Lists (QPLs) may help by shifting the initiative to patients. This study explored dentists’ perceived acceptability of using a QPL to facilitate oral cancer discussions in primary dental care.

**Methods:**

A pre-study patient focus group informed the design. Semi-structured interviews were conducted with 21 primary care dentists working in NHS Scotland. Purposive sampling was used to ensure variation in experience. Interview data were analysed using framework analysis informed by the Theoretical Framework of Acceptability (TFA).

**Results:**

Dentists welcomed the QPL as a valuable, patient-centred tool that could fulfil their ethical duty to inform patients about oral cancer screening. However, significant concerns emerged around time constraints and staffing shortages within high-pressured NHS environments, making any additional intervention feel burdensome. For successful implementation, dentists suggested two prerequisites: (1) design optimisation using short, categorised design with simple language, and (2) systemic integration which was proposed to embed QPLs into booking systems with clear clinical guidelines and systematic training.

**Conclusion:**

While dentists supported QPLs as an acceptable and ethical aid for opening difficult conversations about oral cancer, its successful implementation is contingent upon a user-friendly design of the tool and a systemic integration by addressing the fundamental structural inhibitors of NHS dentistry. Future interventions should focus on integrating QPLs into routine workflows rather than treating them as an add-on task.

**Supplementary Information:**

The online version contains supplementary material available at https://doi.org/10.1186/s12903-026-08911-5.

## Background

Compared with the UK average, Scotland has seen a rapid increase in the incidence and mortality rates of oral cancer with little improvement over the past 40 years [1–3]. The current crisis with limited availability of NHS dental service across the UK may inevitably delay the timely identification of oral cancer [4, 5]. Early detection of oral cancer has been shown as the most effective approach to improving survival, reducing morbidity and hospital costs and improving patient quality of life [3]. This can be seen in better survival rate of 81% for Stage 1 (early disease) patients to 17% for Stage 4 (advanced oral cancer) patients [6]. While not all types of oral cancer can be readily visible during standard oral inspections, visual inspection remains the most common method for early detection of oral cancer [7].

Despite the clear benefits of early diagnosis, patients with oral or oropharyngeal cancer experienced the prolonged delays before seeking a first consultation [8]. Poor public awareness of risk factors, signs and symptoms of oral cancer remains the primary drivers for these delays [9]. While high-risk patients are statistically less likely to attend dental services regularly, the primary care dental team still represents a critical, underutilised resource for opportunistic screening and patient education [10]. Nevertheless, the evidence indicates that dentists often hesitate to initiate these discussions due to concerns about invoking patient anxiety or a perception that patients may be disinterested in receiving this health advice [9, 11].

The perceived barriers to initiating a discussion about oral cancer necessitate interventions to structurally support the dentist-patient communication during routine dental visits. One potential solution is equipping patients with a patient-led agenda to encourage information exchange, enhance health literacy, and promote timely care seeking behaviour. This patient-centred communication aid, known as a Question Prompt List (QPL), consists of a list of health-related questions regarding risk factors, early signs and symptoms that empower patients to proactively ask questions during consultations. The use of QPLs is associated with improved patient recall of information and prompts clinicians to give patients more detailed and personalised information at oncology settings [12, 13]. While QPLs have not been used in primary dental care, a recent study has shown promising outcomes of using a QPL-like pre-appointment questionnaire to help dentists better communicate with anxious children [14].

Before conducting a QPL intervention at primary dental care, we need to understand how dentists perceive the use of this patient-led communication aid within primary dental care settings, as a necessary step in the intervention development process. This aligns with feedback obtained from the PPI advisory group prior to this study (See Methods). Therefore, this study aims to explore dentists’ perceived needs and acceptability of using QPLs to facilitate patient communication during dental appointments. More specifically, the study seeks the dentists’ views on potential facilitators and barriers to using a QPL in their routine practice.

## Methods

### Pre-study meeting with PPI members for project development

A pre-study focus group with patient representatives was held to identify how project aims would best serve the public’s interest and to inform the project development. The pre-study meeting was coordinated by a PPI manager from Dundee University. The proposed idea of a QPL intervention was presented towards four PPI members from the public. Discussion was held between the principal investigator (SY) and the PPI members in terms of the study’s suitability and feasibility. QPLs have been understood by PPI members as an important communication aid to raise oral cancer awareness. However, concerns were expressed about dentists’ perceived acceptability and feasibility of using a QPL to support their communication of oral cancer screening. This has been supported in a systematic review highlighting the importance of clinician acceptance of using a QPL [15]. A preliminary study was therefore suggested to explore dental providers’ needs and buy-in of using a QPL as part of their routine clinical practice, prior to the design of a focused QPL and a future intervention study.

### Study design and ethical considerations

A cross-sectional qualitative study was conducted using semi-structured interviews with dentists working at primary care dental practices in Scotland. Ethical approval was obtained from Dundee University’s Research Ethics Committee (Reference No: UOD-SREC-SDEN-2024–029). The conduct of the study was in accordance with the Declaration of Helsinki. The study adopted the Consolidated criteria for reporting qualitative research (COREQ) with a full checklist (Table S1) [16].

### Participants

Participants were recruited via social media advertisement, through visiting high street dental practices as well as word of mouth. Purposive sampling was used to identify participants for interview to obtain diverse perspectives in their experiences of interest. The research team had no established relationships with the participating dentists. Eligibility criteria included dentists who provide NHS primary dental care services in Scotland. Recruitment took place between January and April of 2025 and was completed when no new themes were derived from the interview content.

A Participant Information Sheet (PIS) was provided to the interested participants, and a completed consent form was sought and signed before the study participation. At the end of the interview, a £25 e-voucher was sent to the participants by email as a token of appreciation for their participation.

### Data collection

Data were collected by the research assistant (XL) through semi-structured interviews. An interview guide with open-ended questions was developed by XL and SY to explore participants’ views on whether a QPL could support the communication with patients to raise patients’ awareness of oral cancer, and how it can be used during routine dental appointments. This interview guide was piloted with two volunteering dentists who were postgraduate research students during the study period to ensure the sequence and the wording of the questions were appropriate to enhance the clarity of the discussion.

Interview questions included:

i) Have you ever heard of a Question Prompt List (QPL)?
ii) What is your general impression of QPL?
iii) What are your thoughts on using a QPL in routine dental practice?
iv) What might be the positive and negative aspects of QPLs from your perspective?
v) Is there anything else you would like to add regarding improving communication about oral cancer to raise patient awareness?

In case participants have no knowledge of what a QPL is, a simplified exemplar of an oncology QPL from the medical literature [17]was supplied to stimulate participants’ responses. Interviews were conducted online, out of NHS work hours, to accommodate the participants’ needs and were recorded via a video conferencing software (TEAMS).

Interview data were transcribed verbatim by the research assistant (XL) and were checked by the principal investigator (SY). Microsoft Excel was used for organising the data. Framework Analysis was adopted using the Theoretical Framework of Acceptability (TFA) [18] to map the data to the seven theoretical domains, namely, Affective Attitudes, Burden, Perceived Effectiveness, Ethicality, Intervention Coherence, Opportunity Costs and Self-efficacy (Table 1). Data were analysed using a predominantly deductive, theory-driven approach guided by the Theoretical Framework of Acceptability (TFA) and developed into three overarching themes.

**Table 1:** TFA Construct {Sekhon, 2017 #17}

TFA Constructs	Definition
Affective Attitudes	How the dentists *feel* about using the QPL
Burden	The dentists perceived amount of effort required to use the QPL
Perceived Effectiveness	How well dentists believe the QPL will achieve its purpose
Ethicality	How well the QPL fits with the dentists’ professional values and ethical beliefs
Intervention Coherence	How well the dentists understand the QPL and how it works
Opportunity Costs	The benefits, profits, or values that must be given up to engage with the QPL
Self-efficacy	The dentists’ confidence in their ability to use the QPL

Data were analysed using the below stages:

1. Data familiarisation
2. Coding data using TFA
3. Data summary based on the TFA construct
4. Developing higher-level themes for data interpretation

Two researchers (XL and SY) read through all the transcripts repeatedly to familiarise themselves with the data (Stage 1). Then they met to discuss TFA domains to ensure that TFA is a suitable framework relevant to the data and then to have a shared understanding of the definitions before they independently coded the data based on TFA matrix (Stage 2). The coding process applied both deductive using TFA as the framework to map original data (first level, Stage 2 and 3), and inductive (second level) to identify higher-level of themes drawn from the seven TFA domains (Stage 4) and to allow the flexibility of developing any new themes organically from the original data.

The seven constructs of the TFA framework were used to guide the qualitative summaries and development of sub-themes, bringing together key features of the data and interpreting them. Meetings were held iteratively when XL and SY completed their initial independent coding using TFA matrix to ensure consistency and rigour for data analysis [18]. Both main coders (XL and SY, both female) had qualitative research experience with the latter (SY) having nearly 20 years of research experience as a senior researcher. More importantly, both researchers had dental professional background overseas but trained in the UK as dental public health researchers. This combination of insider and outsider’s perspectives allowed the researchers to have in-depth understanding of the data but also maintain a relatively objective perspective to interpret the findings [19]. Theme development was carried out and discussed between XL and SY to ensure clarity and consistency (Stage 4). The overall themes and findings were then reviewed by the wider team (including GH and TN) to enhance the trustworthiness and credibility of the analysis [20]. While clinical time constraints prevented the formal return of all full transcripts to participants for correction, we invited all participants to review our preliminary thematic mapping to enhance trustworthiness [20]. A self-selected sub-group of participants responded and confirmed that our interpretations accurately reflected their perspectives.

## Results

The interview data were mapped based on the TFA construct and the main themes were developed as follows: (1) Perceived value in facilitating patient-centred communication; (2) Systemic barriers to implementation; (3) Prerequisites for implementation: Design optimisation and system integration. A summary table with themes and explanations was presented in Table 2.

**Table 2:** A summary of themes with explanations

Themes	Explanations
1. Perceived value in facilitating patient-centred communication	The dentists valued the structured, patient-led nature of the QPL which could empower patients to proactively initiate sensitive oral cancer prevention discussions and select relevant topics. This also helps fulfil their duty as a dental health professional
2. Systemic barriers to implementation	Severe time constraints within treatment-oriented appointments and restricted staffing capacity represent the main structural barriers to embedding the QPL intervention within busy NHS practice
3. Prerequisite for implementation: Design optimisation and system integration	Feasibility of the QPL intervention relies on simplified and user-friendly design to minimise patient cognitive load, and wider system integration to adopt the tool into routine clinical workflows, online booking systems, professional guidelines and dental education

### Sample characteristics

Out of 25 dentists being approached, 21 primary care dentists took part in the semi-structured interview with 18 from the General Dental Services (GDS, i.e. high street dental practices providing NHS primary dental care) and 3 from the Public Dental Services (PDS, i.e. NHS dental services delivered in community settings, often for patients with additional care needs or barriers to accessing general dental services). We did not receive further feedback from the four non-respondent dentists. Participants included a balanced mix of genders (10 males and 11 females) with a range of clinical qualified experiences from 2 years to 10 + years (Table 3). Interviews lasted between 16 and 35 min.

**Table 3:** Characteristics of the participants

Participant	Gender	Year of qualification	Type of primary dental care	Interview length
1	Female	2 years	GDS	35 min
2	Male	2 years	GDS	26 min
3	Female	3 years	GDS	22 min
4	Male	5 years	GDS	25 min
5	Male	10 years	GDS	20 min
6	Male	7 years	GDS	20 min
7	Female	5 years	GDS	22 min
8	Male	5 years	GDS	25 min
9	Male	6 years	GDS	27 min
10	Male	10 years	PDS	20 min
11	Male	2 years	GDS	25 min
12	Female	6 years	PDS	18 min
13	Female	5 years	GDS	22 min
14	Female	2 years	GDS	28 min
15	Female	2 years	GDS	26 min
16	Female	4 years	GDS	25 min
17	Female	4 years	GDS	22 min
18	Female	6 years	GDS	20 min
19	Male	7 years	GDS	25 min
20	Male	8 years	GDS	30 min
21	Female	17 years	PDS	22 min

*GDS* General dental services, *PDS* Public dental services

#### Theme 1. Perceived value in facilitating patient-centred communication

Most participants welcomed the idea of having QPL as a communication aid to initiate this difficult conversation. Even though they agreed that cancer could be a ‘scary word’, many participating dentists thought they had ethical duty to inform patients of what examinations they were doing and more importantly why they do it. Such strong moral obligation to provide transparent care was captured as reflected in the below extract:

*“Cancer can be a scary word, but it is one we probably need to use…. We probably have a duty to make them aware of that. We do oral cancer checks and why we do them.”* *(Interview 12, 5 years)*

This perspective highlights a positive Affective Attitude toward the QPL. While acknowledging the discomfort to mention the word "cancer," the participant prioritised patient awareness and regarded the tool as an ethically necessary way to bridge this difficult conversation.

While none of the dentists had previously used QPL, they seemed to have a good understanding of how QPLs can work well in routine dental checkup. The value of ‘patient-centred’ communication within QPL has been highlighted in the below extract by a dentist:

*“…so with the QPL, we have a clear idea about what the patients want to (discuss) and also make it easier to speak to the patients, because they already prepared their mind to discuss the questions with us.”* *(Interview 5, 3 years)*

This quote showed that the list has been understood by the dentist as a tool that could not just give them a ‘clear idea’ of what patients want to discuss, but also had ‘patient-led’ functions that could empower patients to proactively select oral health related topics to discuss with their dentist.

While most of the participants accepted the idea of integrating the QPL, a different view emerged regarding the concerns of its potential to disrupt the rapport with patients. One experienced dentist has shown her concern whether the structured nature of the list would affect the natural flow of the conversation:

“*I worry that if we just go through a checklist, we lose the natural flow of the conversation."* (Interview 7, 10 years).

This reflected a minority of dentists who would prioritise the organic patient rapport and the flexibility of interacting with their patients. This emphasised the training needs from dental professionals on how to use the QPL flexibly without disrupting the natural dentist-patient interaction.

#### Theme 2: systemic barriers for implementation

Time constraints have been identified as the main burden for implementation. The dentists described the current NHS dentistry environment as ‘high pressured’ (Interview 7, 10 years) and ‘high demand’ (Interview 9, 6 years) with ‘high volume’ of patients (Interview 2, 2 years). Therefore, most dentists were concerned that the implementation of the oral cancer communication would be challenging within their busy practices and limited appointment schedules. This inevitably adds ‘burden’ to their existing stretched workload. This concern has been highlighted by one experienced dentist who stated that:

*“Even with a structured tool, fitting an in-depth discussion about oral cancer into a standard appointment could be difficult, especially in a busy practice.”*

*(Interview 10, 10 years)*

Participants also highlighted that introducing QPLs could increase the workload for dental teams by requiring additional staff support. Without adequate staff to assist patients in completing and processing the QPLs, dentists feared that the tool would disrupt clinic flow and increase administrative pressures.

*" We need staff to organise the patients to fill up the QPLs and also to explain something to them and then collect their paper and deliver that to the dental staff…”*

*(Interview 4, 7 years)*

#### Theme 3. Prerequisite for implementation: optimisation and integration

[1] Optimisation with user-friendly design

Despite time constraints as the main barrier in a ‘busy’ NHS practice, many participants suggested possible solutions to a smooth implementation of QPL. One key solution was to simplify the design to enhance usability and reduce cognitive load during consultation. Participants emphasised that a clear and well-structured design would be necessary to achieve the intervention coherence. This practical design idea has been discussed by one participant who gave a direct strategy for optimising the usability of the tool:

*"(We need to) make it shorter, structured, like divide them into categories… Don’t make it to be too complicated. Make it to be as simple as possible."*

*(Interview 19, 3 years)*

By adopting a streamlined and categorised design, the participant highlighted how the design choices could alleviate the perceived burden of the consultation, and to ensure the list is accessible for patients without compromising the limited appointment time.

Several participants highlighted the importance of tailoring QPLs to different audiences rather than adopting a one-size-fits-all approach. They felt that personalisation would make the tool more relevant and engaging for patients, as well as more manageable within the primary dental setting. Suggestions included making the QPL ‘short’, categorising questions by theme and using ‘easy’ language.

*"…The language we use with (patients from) different backgrounds, so it might not be easy for everyone to understand. Maybe the question would be best to be used (in) easy*

*language to be understandable by everyone with different backgrounds."*

*(Interview 17, 6 years)*

In addition to the structural simplicity, an additional minor theme emerged regarding the language adaptability of the tool. A participant highlighted that the tool should be made available in multiple languages to accommodate the diverse patient populations:

*“I think providing more languages is good. Don’t make it to be too complicated.”*

*(Interview 19, 3 years).*

Such perspective underscores the diverse needs from patients from different cultural and language backgrounds, which is essential to ensure the equitable accessibility of health information to reach the whole population.

[2] System integration.

Most dentists recognised the importance of systemic integration of the QPL into the wider clinical system. They thought QPL should be introduced as a standardised procedure when the exam appointment was booked.

*“I think the first thing would be to introduce it into the exam system. So it’s part of the normal exam or the appointment. So we don’t think it is separately with our routine work*.” (Interview 20, 8 years).

The effective use of QPL requires a coordinated joint effort that needs teamwork. The below excerpt illustrates the QPL’s journey from the receptionist to dentist and then for record keeping. If there is a failure at any point of this chain, this can result in a breakdown of the whole system:

*“the patients come there, and they get it. And so the receptionist needs to know the process first...…then they may be handed to the nurses or the dentists... after the communication finishes, the documents need to be noted or stored... So I think that requires the teamwork from the whole dental team.”*

(Interview 8, 7 years)

To support such integrated workflow using team-based approach, participants also found other enabling factors such as systemic training which not just involved continued professional development but also to include undergraduate education throughout a dentist's career to ensure dentists know how to introduce and use the QPL effectively. On the other hand, clinical guidelines and protocols have been cited by dentists as another enabler so that they feel more confident and would know when and how to introduce the QPL, especially in time-limited appointments.

## Discussion

To our knowledge, this study was the first to introduce Question Prompt Lists (QPLs) as a potential communication intervention tool to improve oral cancer conversation at primary dental care. Our study aimed to explore dentists’ buy-in of using a QPL as a structured communication tool to facilitate discussing the sensitive oral cancer topic at routine dental checkup. This study found that while Scottish primary care dentists perceive QPLs as an acceptable and valuable tool for initiating oral cancer discussions, the implementation appears to be primarily restricted by systemic barriers within NHS dentistry, particularly limited time and staffing capacity. Feasibility was seen as contingent upon both design optimisation and systemic integration.

Question Prompt Lists have been predominantly used in oncology and palliative care consultations [15], yet there is an international trend with successes shown in transitioning from oncology into primary care and chronic disease management [13, 21, 22]. Our study, although exploring dentists’ views of a hypothetical QPL in primary dental care, revealed that dentists favoured its potential to resolve the discomfort of bringing up a sensitive topic of oral cancer by shifting the initiative to patients. Such high perceived value of the QPLs among dentists addresses a notable gap in the recent literature which lacked empirical evidence regarding clinicians’ perspectives about the benefits of such tools [23]. Moreover, the ‘patient-led’ function (Interview 10 and 13) was valued by our dentists not just as convenience, but to empower patients and make the discussion feel more natural and invited, which resonates with recent evidence showing QPL was valued by other health professionals in engaging patients in meaningful conversations with sensitive topics [24]. More importantly, dentists in our study perceived the QPL as an *effective* ‘conversation starter’ (Interview 13) which could transition the consultation from traditional one-way advice giving to a dynamic ‘two-way’ exchange (Interview 21). Thus, by providing such a structured communication guide to the difficult talk, the QPL allows for the endorsement of shared decision making within time-pressured dental context [25, 26]. Furthermore, the perceived value of QPLs among the participating dentists in our study has added evidence to a recent systematic review, which highlighted a lack of evidence regarding clinicians' perspectives on the benefits of these tools [15].

Many participants felt they had an ethical duty to inform patients that a check-up includes oral soft tissue examination, and more importantly, to explain the rationale behind it. The QPL was seen as instrumental in providing a structured yet patient-led approach to fulfilling dentists’ ethical duty, particularly for vulnerable groups such as patients with poor health literacy or those at higher risk. This is in line with another qualitative study that doctors feel they are ‘responsible’ to encourage their patients to ask questions [27].

Time constraints and lack of staffing were cited as the contextual barriers that makes any additional intervention feel burdensome. Although dentists valued the concept of QPL, they anticipated the potentially related workload within the real-world constraints of NHS dentistry. Participants consistently described the dental appointment as ‘tight’ or ‘high pressurised’. The introduction of a QPL was perceived not as an integrated activity, but as an added task that threatened to extend consultations or reduce their time for essential care. This concern directly impacts dentists perceived *self-efficacy* as they questioned their ability to effectively use the tool without disrupting the normative clinic flow. This mirrored the broader NHS literature identifying time and resource constraints as fundamental barriers to adopting new practices [28, 29]. Some dentists were worried that handing patients a list of questions could lead to unpredictable conversations, potentially opening complex or distressing topics they might not feel confident to manage within a routine check-up. This finding was consistent with the concerns from other healthcare settings where clinicians feared QPLs might reduce their control over consultation dynamics [30]. This highlights a critical tension: while the QPL was seen as *ethically* aligned and *effective* in theory, its *burden* in practice was viewed as potentially overwhelming.

Despite time constraints, the participants proposed design optimisation (at micro level) and systemic integration (at macro level) as interdependent prerequisites for feasible implementation. At the micro level, they highlighted the importance of a user-friendly design for feasibility. A simple and user-friendly design of the question list can help alleviate potential cognitive load for patients, as the tool itself could overwhelm patients—particularly in an anxiety-provoking environment like a dental surgery. Therefore, co-designing a simple, accessible QPL is essential to ensure its *affective acceptability* for all users. The success of co-designing a QPL in implementation has been evidenced in other healthcare settings such as antenatal and paediatric clinics [31, 32].

Nevertheless, participants consistently argued that design optimisation alone would be insufficient. The main solution focused on systemic integration to reduce the perceived *burden*. They thought the QPL had to be embedded within existing systems to become a sustainable and normalised part of the clinical practice. This spans from clinical guidelines, dental education and Continuing Professional Development (CPD) to integration with online booking systems that prompt its use. Such call for system-level change aligns strongly with implementation science theory, i.e. the Normalisation Process Theory, which posits that for a complex intervention to become ‘normalised’, it must be supported by collective action (e.g., whole-practice adoption, training) and embedded in existing workflows and structures [33]. To effectively overcome the barriers of time and staffing constraints, the QPL must be embedded as a standardised procedure through providing essential legitimacy, training, and peer support, which collectively build user self-efficacy. This shift from an add-on to an integrated component is fundamental to transforming prospective acceptability into successful, sustained implementation.

### Strength and weaknesses: trustworthiness and limitations

Our study is the first study to explore dentists’ views of using QPL as a potential intervention to help with a sensitive oral health related topic – oral cancer. The involvement of PPI group at the planning stage of the project adds relevance and urgency. Moreover, we have used TFA as a theoretical framework to map our findings and developed high level themes based on its key constructs across participants. This deductive-inductive hybrid strategy has avoided the rigid use of merely deductive coding and ensured the findings are both theoretically grounded and data-driven. To promote trustworthiness, we have refined our findings for thematic mapping by checking with some of the participants during the analysis stage [20, 34].

This study had several limitations. First, this study did not have wide representation given its qualitative nature characterised by a relatively small sample size. Second, there might be selection bias due to those who rejected to participate in the study. Furthermore, the participant validation process relied on a self-selected subgroup of participants who responded to our invitation to review the findings. Finally, the study was conducted with dentists working in Scotland which may have limited generalisability to other contexts.

## Conclusion

While dentists perceived the QPL as an ethically sound and effective tool to facilitate patient-led oral cancer discussions, they were concerned about its implementation which might be restricted by structural inhibitors within NHS dentistry. These structural barriers include time constraints during treatment-oriented appointments and limited staffing capacity to manage additional intervention tasks. Such capacity might affect equitable service delivery, particularly for high-risk or low-literacy populations experiencing the greatest systemic barriers to accessing primary dental care.

Nevertheless, successful implementation can be achieved through design optimisation to minimise patient cognitive load, and systemic integration spanning from clear clinical guidelines, education and training, and embedding the tool directly into standard booking workflows. Future interventions using QPLs should consider these systemic factors to encourage implementation success.

## Supplementary Information

Supplementary Material 1.

## Data Availability

The datasets generated and/or analysed during the current study are not publicly available due to the potential risk of participant identification but are available from the corresponding author on reasonable request.

## Abbreviations

CPD: Continuing Professional Development
GDS: General Dental Service
PDS: Public Dental Service
NHS: National Health Service
PPI: Patient and Public Involvement
QPL: Question Prompt List
TFA: The Theoretical Framework of Acceptability
